# Infective Endocarditis Due to Serratia odorifera: A Case Report and Literature Review

**DOI:** 10.7759/cureus.52640

**Published:** 2024-01-20

**Authors:** Muhammad Hussain, Bereket Tewoldemedhin, Muhammad Waqas, FNU Marium, Nardos Tewoldemedhin, Siham Hussien, Miriam Michael, Jihad Slim

**Affiliations:** 1 Medical Education, Saint Michael's Medical Center/New York Medical Center, Newark, USA; 2 Internal Medicine, Suburban Community Hospital, Lower Bucks Hospital, Bristol, USA; 3 Infectious Diseases, Saint Michael's Medical Center, Newark, USA; 4 Internal Medicine, Saint Michael's Medical Center, Newark, USA; 5 Internal Medicine, College of Health Sciences, Addis Ababa University, Addis Ababa, ETH; 6 Internal Medicine, University of Maryland Medical Center Midtown Campus, Baltimore, USA; 7 Internal Medicine, Howard University, Washington DC, USA

**Keywords:** av graft, transesophageal echocardiography (tee), hemodialysis, infective endocarditis, odorifera, serratia

## Abstract

*Serratia odorifera* from the time of its discovery in the 1970's had been considered a common colonizer of the skin with little pathogenic potential. Cases of human infections caused by *S.* *odorifera *are relatively rare. To date, very few cases have been reported describing primarily bloodstream and urinary tract infections.

We describe a patient who developed endocarditis due to *S.* *odorifera* confirmed with a transesophageal echocardiogram. The patient was treated with six weeks of antibiotics with uneventful recovery. After a thorough review of published literature, we concluded that this is the first case of endocarditis caused by *S. odorifera *reported in English literature.

## Introduction

*Serratia odorifera* belongs to the Enterobacteriaceae family first described by Grimont et al. in 1978 as a new species of Serratia with two unique biotypes that have differing biochemical characteristics with regards to growth on sucrose and ornithine [1,2]. It is named *S. odorifera* due to the typical odor it produces in cultures. It had initially been considered a common saprophyte colonizing the human skin and in food [2]. The significance of *S. odorifera* as a human pathogen was unclear until the first documented bacteremia from *S. odorifera* in 1987 [2,3]. The cases of human infection associated with *S. odorifera* are relatively uncommon with few reports of catheter-associated infections [3,4]. After an extensive review of English literature, we have concluded that this is the first reported case of endocarditis caused by *S. odorifera.*

## Case presentation

A 60-year-old male with a past medical history of hypertension, congestive heart failure with preserved ejection fraction, end-stage renal disease on hemodialysis for 20 years, with an underlying history of Conn’s syndrome presented to the emergency department with a complaint of fever and generalized fatigue for two days. His symptoms were associated with chills and rigor for the same duration. He had also endorsed progressive shortness of breath with exertion worsening over the same period. The patient had no palpitations, chest pain, abdominal complaints, or urinary complaints. He denied any history of intravenous drug use or smoking. He stated to be compliant with his dialysis schedule and had an uneventful session on the morning of his symptom onset. The patient had a polytetrafluoroethylene (PTFE) AV graft on the left upper extremity for hemodialysis access placed seven weeks prior to his presentation. The graft had been in use for the past three weeks with no difficulty.

His vital signs at presentation were as follows: temperature was 101.9°F, heart rate 93 beats/minute, blood pressure 150/74 mmHg, respiratory rate 22 breaths/min, and oxygen saturation of 95% on room air. The physical examination revealed mild conjunctival pallor. The left forearm AV fistula site was clean with good palpable blood flow. Laboratory results showed elevated white blood cell (WBC) count with neutrophil predominance and low hemoglobin. Serum creatinine was elevated in keeping with his end-stage renal disease but with normal blood urea nitrogen. The rest of the electrolytes were unremarkable (Table 1).

**Table 1:** Laboratory results of the patient.

Parameters	Results	Reference values
Hemoglobin	10.4 g/dL	12-16 g/dL
Platelets	221,000/µL	150,000-400,000/µL
White blood cell	11,900/µL	3,100-10,500/µL
Neutrophils	10,400/µL	1,500-7,000/µL
Neutrophils (relative percent)	88%	40-60%
Lymphocytes	300/µL	1,000-4,000/µL
Lymphocytes (relative percent)	2.6%	20-40%
Monocytes	600/µL	300-900/µL
Monocytes (relative percent)	5.5%	4-8%
Aspartate transaminase	31 IU/L	10-36 U/L
Erythrocyte sedimentation rate	132 mm/h	0 - 20 mm/h
C-reactive protein	9.9 mg/dL	0.0-0.8 mg/dL
Glucose	100 mg/dL	70-140 mg/dL
Blood urea nitrogen	38 mg/dL	6.0-24 mg/dL
Creatinine	9.9 mg/dL	0.5-1.0 mg/dL
Sodium	136 mmol/L	136-145 mmol/L
Potassium	4.9 mmol/L	3.5-5.3 mmol/L
Chloride	108 mmol/L	98-110 mmol/L
Albumin	3.5 g/dL	3.6-5.1 g/dL

**Figure 1:**
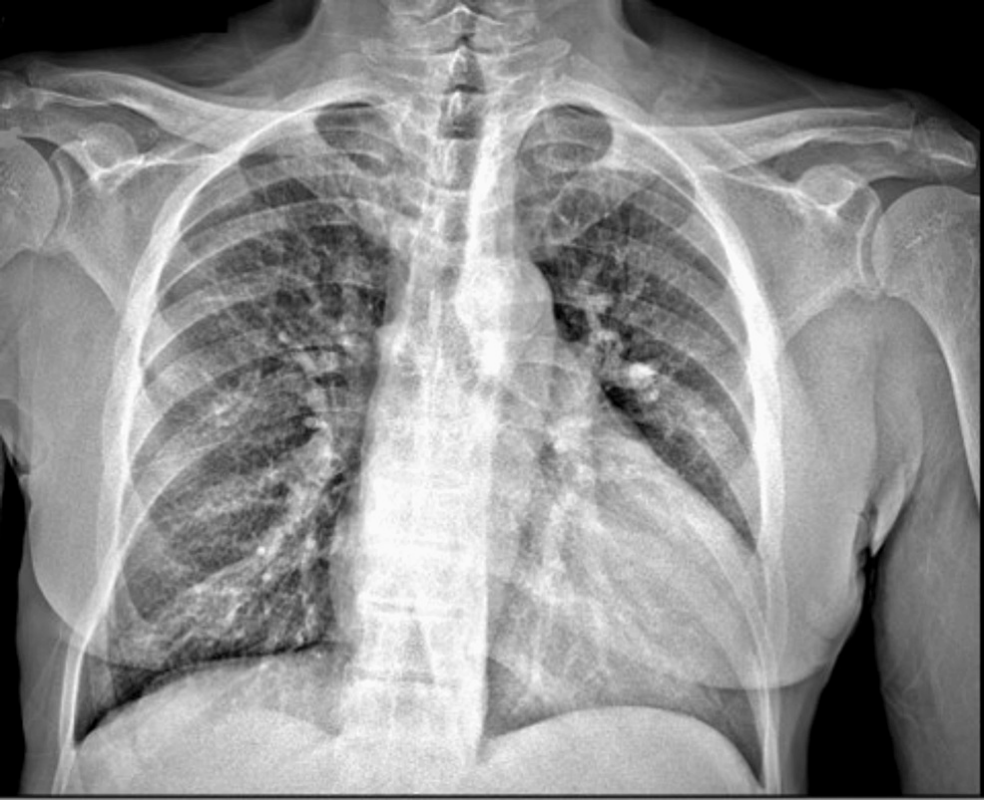
Chest x-ray showing cardiomegaly with non-specific perihilar prominence.

Two sets of blood cultures were obtained on admission which grew two of two bottles of Gram-negative rods that were identified as *S. odorifera* (Table 2). An electrocardiogram (ECG) revealed a sinus rhythm with a normal PR interval. Chest x-ray showed non-specific airspace and interstitial abnormalities with perihilar predominance. Stable cardiomegaly was noted (Figure 1).

**Table 2:** Antimicrobial susceptibilities for Serratia odorifera from blood culture, determined based on minimum inhibitory concentration (MIC).

Antibiotic	MIC (mg/L)	Interpretation
Ampicillin/sulbactam	>16/8	Resistance
Cefepime	>16	Resistance
Ceftazidime	>16	Resistance
Ceftriaxone	>32	Resistance
Ciprofloxacin	≤1	Sensitive
Gentamicin	≤4	Sensitive
Meropenem	≤1	Sensitive
Piperacillin/tazobactam	>64	Resistance
Tobramycin	≤4	Sensitive
Trimethoprim/sulfamethoxazole	≤2/38	Sensitive

**Figure 2:**
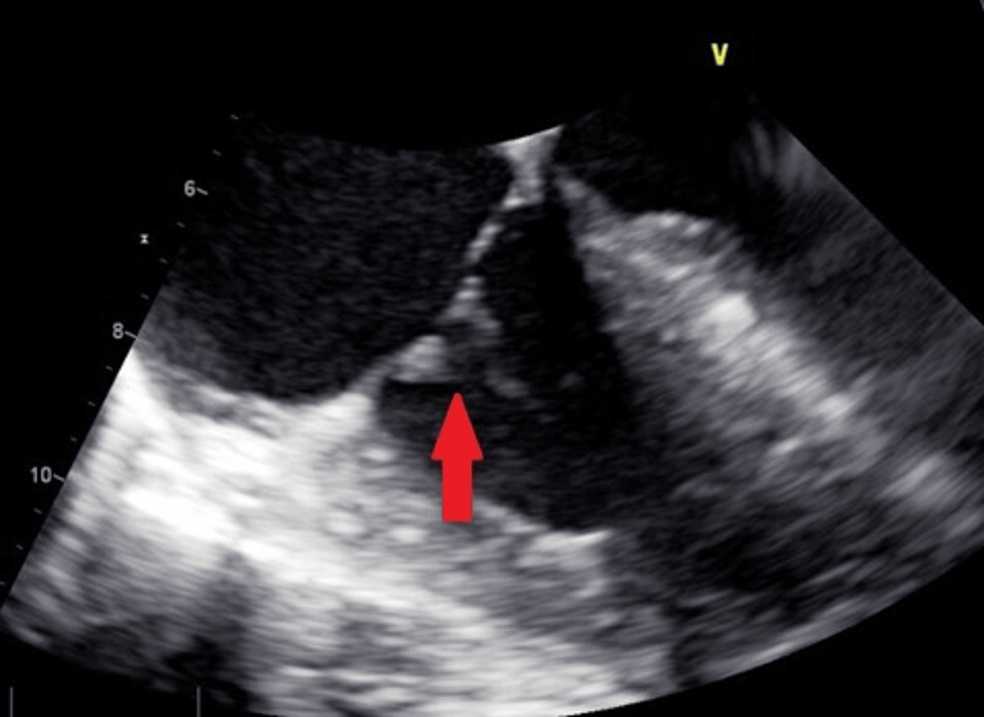
Transesophageal echocardiogram (TEE) image demonstrating a 0.9x0.6 cm vegetation attached to the septal annulus of the tricuspid valve (arrow).

Computed tomography (CT) scan abdomen and pelvis were negative for acute abdominal pathology. Duplex examination of the AV graft showed no technical defects in anastomosis and there was no evidence of perigraft fluid. MRI thoracic and lumbar spine were negative for extradural and subdural abscess. Transthoracic echocardiogram was unrevealing. Transesophageal echocardiography (TEE) was pursued which revealed small vegetations less than 1 cm attached to the septal tricuspid valve leaflet with no evidence of abscess or significant valve dysfunction (Figure 2).

A definite diagnosis of infective endocarditis was established with two major Duke-modified criteria met. Antibiotics were subsequently streamlined to meropenem with amikacin adjusted for dialysis in milieu of infective endocarditis due to a possibility of AmpC beta-lactamase-producing Serratia based on the susceptibility pattern observed. Aminoglycoside was discontinued after two weeks of therapy to minimize adverse drug reactions. The patient remained afebrile with the resolution of his leukocytosis on the second day of effective therapy. His blood culture was negative on the repeat set of cultures taken 48 hours after the initial set.

The duration of carbapenem was determined to be six weeks for effective treatment of infective endocarditis with non*-*Haemophilus species, Aggregatibacter species,* Cardiobacterium hominis*,* Eikenella corrodens*,and Kingella species (HACEK) group Gram-negative bacillus. He continued to undergo hemodialysis as per schedule. The clinical condition of the patient improved during the hospital stay and he tolerated the treatment regimen well.

The patient was discharged to a skilled nursing facility to complete intravenous antibiotic therapy and to continue his dialysis sessions. He had an uneventful recovery after the completion of six weeks of antibiotic therapy. Further outpatient management involved a multidisciplinary team consisting of nephrology, cardiology, and infectious disease in addition to primary care.

## Discussion

Serratiais a genus of Gram-negative rod-shaped bacterium, belonging to the Enterobacteriaceae family, with at least 14 different related species [1,2]. The most common species as a cause of human disease in this genus is *S. marcescens* [1,2].

Serratiaorganisms are known to cause serious and often fatal Gram-negative infections in humans [1,2]. Most Serratia infections result from nosocomial infections with the portal of entry being the respiratory or urinary tract [2,3]. Severe infections are usually seen in patients with previous antibiotic therapy, neutropenia, underlying hematologic malignancies, chronic underlying severe liver disease, severe renal failure, and patients on long-term hemodialysis [2-4]. Prognosis usually correlates with the patient's overall medical condition and the rapidity of treatment institution, with mortalities approaching 64% if untreated [2-4].

*S. odorifera* was first identified as a new species of Serratia in the late 1970s [2-4]. Although the habitat remains to be fully specified, isolates have been recovered from plant sources [3]. Two biogroups have been identified with the distinction being made based on their ability to ferment sucrose and raffinose and their ability to decarboxylase ornithine [4,5]. The importance of this biogrouping of *S. odorifera* is still poorly understood, but to date, documented human infections have been more associated with biogroup 2 with isolates being found in blood and CSF [3,4]. In contrast, most isolates of biogroup 1 are cultured as saprophytic growth within the respiratory tract [3,4].

Infective endocarditis (IE) remains among the top four leading causes of life-threatening infections along with sepsis, pneumonia, and intraabdominal abscess [5]. The landscape of diagnosis and therapeutics of IE continues to evolve as patient demographics change, with more invasive endovascular procedures and instrumentations [5,6]. The microorganisms typically associated with endovascular infections continue to change with more nosocomial and resistant organisms emerging [5]. Risk factors for IE include cardiac factors (prior history of IE, structural heart disease, valvular disease, congenital heart disease, intracardiac device), individual factors (such as intravascular device, intravenous drug abuse, poor dentition, immunosuppression, or chronic hemodialysis), or recent dental and surgical procedures [5,6]. Clinical manifestations can be variable consisting of constitutional symptoms like fever, chills, arthralgia, anorexia, malaise, headache, night sweats, abdominal pain, dyspnea, cough, and pleuritic pain. The diagnosis of IE is made using the modified Duke criteria, which was initially developed as a research tool to standardize the protean clinical manifestations associated with IE and has been undergoing constant revision and modification with the most recent update in 2023 by the International Society of Cardiovascular Infectious Disease [5,6]. The recently modified criteria incorporate pathological criteria which encompass intraoperative inspection of the valves; microbiological criteria which have been updated to include diagnostics with enzyme immunoassay techniques, polymerase chain reaction, metagenomic sequencing, and in situ hybridization; imaging criteria which in addition to echocardiography, now include positron emission computed tomography with 18F fluorodeoxyglucose and cardiac computed tomography [5,6]. The incidence of IE in hemodialysis patients has been estimated to be 308 per 100,000 patient-years, which is significantly higher than the general population possibly due to intravascular access and immunosuppression [6,7]. A retrospective study involving 296 patients with a diagnosis of IE with 52 on hemodialysis revealed that the most involved valve was the mitral valve (55.8%) followed by the aortic (21.7%) [7]. The most commonly isolated organism was *Staphylococcus aureus* in 40% followed by Enterococcus species in 13.7%, with the remaining being a mix of polymicrobial and coagulase-negative staphylococcal species [7].

An extensive literature review on PubMed for English publications with key terms “*Serratia odorifera,*” “infective endocarditis,” and “bacteremia” yielded a total of 12 cases [8-11]. Eight of the 12 reported cases were in neonates in Johannesburg, South Africa, following contaminated parenteral fluid nutrition therapy [8]. The consequence was fatal septicemia in all eight neonates [8]. Among the four adults that had *S. odorifera* bacteremia, three of the patients had a source of infection identified as catheter-associated urinary tract infection which had developed following urinary catheterization for surgical procedures [9-12]. All three patients had chronic renal failure but were still producing urine and were acutely placed on hemodialysis following surgical complications with acute worsening of azotemia [10-12]. Two of the patients had liver cirrhosis [10-12]. Of these patients, one succumbed to the overwhelming Gram-negative sepsis [9,10]. The fourth patient had chronic renal failure with thrombocytopenia and nosocomial sepsis with *S. odorifera* bacteremia developing following the insertion of a Swan-Ganz catheter [11]. The patient was able to recover after effective Gram-negative therapy [11,12]. In our search, we did not find documented cases of *S. odorifera* associated with infective endocarditis.

To the best of our knowledge, there are no prior reported cases of infective endocarditis from *S. odorifera*. The risk factors for endocarditis in our patient could be the intravascular AV graft placed seven weeks prior and hemodialysis. Although the initial source for the bacteremia was not clearly identified, one potential cause could be an already contaminated AV graft or possibly contamination during use which resulted in the tricuspid valve seeding. This study illustrates the potential emerging occurrence of cases of *S. odorifera* bacteremia with endocarditis following placement of polytetrafluoroethylene AV grafts.

## Conclusions

This study illustrates the emergence of *S. odorifera* as another pathogen for infective endocarditis after polytetrafluoroethylene AV graft placement for hemodialysis. We should include this organism in the differential diagnosis of infective endocarditis and the present case serves as a potential benchmark for further research and investigation into the organism and its relationship with polytetrafluoroethylene grafts. This study is the first documented case of infective endocarditis due to *S. odorifera*.
